# Staging of T2 and T3 nasopharyngeal carcinoma: Proposed modifications for improving the current AJCC staging system

**DOI:** 10.1002/cam4.3328

**Published:** 2020-09-01

**Authors:** Chunyan Cui, Haojiang Li, Huali Ma, Annan Dong, Fei Xie, Shaobo Liang, Li Li, Jian Zhou, Chuanbo Xie, Yue Yan, Lizhi Liu

**Affiliations:** ^1^ Department of Radiology Sun Yat‐Sen University Cancer Center State Key Laboratory of Oncology in South China Collaborative Innovation Center for Cancer Medicine Guangdong Key Laboratory of Nasopharyngeal Carcinoma Diagnosis and Therapy Guangzhou P. R. China; ^2^ Department of Radiation Oncology Cancer Center The First People's Hospital of Foshan Affiliated to Sun Yat‐sen University Foshan P. R. China; ^3^ Cancer Prevention Center Sun Yat‐Sen University Cancer Center State Key Laboratory of Oncology in South China Collaborative Innovation Center for Cancer Medicine Guangdong Key Laboratory of Nasopharyngeal Carcinoma Diagnosis and Therapy Guangzhou P. R. China

**Keywords:** AJCC stage system, MRI imaging, nasopharyngeal carcinoma, survival analysis, T staging

## Abstract

**Objectives:**

We aimed to reconstitute T2 and T3 stage classification in nasopharyngeal carcinoma (NPC) cases and verify its utility in clinical settings.

**Materials and Methods:**

We enrolled 792 NPC patients. Cox proportional hazards model was used to compare the effect sizes (hazard ratio [HR]) of the cranial structure invasion on survival and select the structures for up‐staging or downstaging T2 and T3 NPC. The samples were reclassified and the survival curves for T2 and T3 stages were analyzed. The proposed new staging system was validated on an external sample (n = 433).

**Results:**

Thirteen cranial structures were examined. American Joint Committee on Cancer (AJCC) T3 stage patients with the invasion of the base of the sphenoid (HR = 2.58, 95% CI = 1.16‐5.77) or base of the pterygoid (HR = 2.00, 95% CI = 0.84‐4.77) had significantly lower hazard ratios than T2 stage patients with the invasion of soft tissues in the bilateral parapharyngeal space (HR = 5.26, 95% CI = 2.02‐13.68) and single/bilateral carotid sheath (HR = 7.78, 95% CI = 3.06‐19.76). T3 stage with the invasion of the above‐mentioned bones was reclassified as T2, and T2 stage with the invasion of the above‐mentioned soft‐tissue structures was reclassified as T3. Survival analysis showed a significant difference between the reclassified T2 and T3 stages (*P* < 0.001). The results were replicated in the validation samples.

**Conclusion:**

The proposed staging system for defining T2 and T3 stage NPC appears to be superior to the AJCC 8th edition. It could improve prognosis and optimize the treatment selection.

## Graphical abstract

Hazard ratio was lower for AJCC T3 with bone than AJCC T2 with soft tissue invasion ·The 5‐year OS and PFS were higher for re‐classified T2 than T3 stage NPC patients·Modified staging system for T2 and T3 NPC may result in better management.

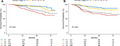

## 1 INTRODUCTION

Nasopharyngeal carcinoma (NPC) is one of the commonest malignant diseases in southern China, with an annual incidence of approximately 30/100 000 persons.^1^ The choice of appropriate treatment for NPC depends on accurate T staging of cancer. A major problem with the 7th and the current 8th version of the American Joint Committee on Cancer (AJCC) staging systems is that T2 and T3 stage NPC patients show comparable survival.^2^, ^3^ The overlapping of the survival curves for AJCC T2 and T3 stage NPC could possibly be due to the following reasons. First, the current 8th version of the AJCC staging system distinguishes T2 and T3 stage NPC cases according to whether or not a tumor invades the cranial bony structure. Specifically, NPC with the invasion of only the parapharyngeal space or nearby soft tissues (ie, medial pterygoid, lateral pterygoid, or prevertebral muscles) is classified as T2 stage, while a tumor invading any of the bony structures of the skull base or cervical vertebrae and/or paranasal sinuses is classified as T3 stage.^4^, ^5^ However, studies have consistently shown that invasion of different bony skull‐base structures had widely differing effects on the long‐term survival of NPC patients.^4^, ^5^, ^6^, ^7^, ^8^, ^9^, ^10^, ^11^ It is possible that NPC patients with the invasion of some specific bony structures may actually have better survival than patients with the invasion of parapharyngeal space or nearby soft tissues. If that is the case, patients with the invasion of these bony structures should be classified as T2 stage patients and those with parapharyngeal space invasion as T3 stage patients. Second, all the structures used for defining T2 and T3 stages in the AJCC 8th version staging system are derived from the reviews of the literature^12^, ^13^, ^14^ and it is highly likely that some structures (eg, the carotid sheath) that are important in distinguishing between T2 and T3 stage NPC cases were missed.

The aim of this study was to develop and validate a more efficient system for staging T2 and T3 NPC, by moving some of the T3 stage NPC cases with a minor invasion of certain specific bony structures into T2 stage and some T2 stage cases with the invasion of soft tissues into T3 stage. In addition, we aimed to find new anatomic structures that were not included in the 8th version of the AJCC staging system but that may add an important prognostic value to the recommended new staging system. We expect that this modified staging system for T2 and T3 NPC will help in a more accurate prognosis and the selection of appropriate treatments.

## 2 MATERIALS AND METHODS

### 2.1 Training and validation samples

The training sample comprised 792 NPC patients treated at the Sun Yat‐sen University Cancer Center between January 2010 and January 2013. Only patients with pathologically confirmed squamous‐cell carcinoma, without distant metastasis, were included. Those without complete magnetic resonance imaging (MRI) data or information on hepatitis B virus (HBV) or Epstein‐Barr virus (EBV) loads were excluded. Figure S1 shows the training sample selection process. The validation sample comprised 433 NPC patients treated at the First People's Hospital of Foshan between April 2010 and March 2014. The inclusion and exclusion criteria were the same as for the training sample.

This study was approved by the Institutional Review Boards at Sun Yat‐sen University Cancer Center and the First People's Hospital of Foshan. All participants provided written informed consent for participation in the study.

### 2.2 MRI examination

MRI scans of the head and neck regions (ie, from the saddle pool to the lower edge of the sternal end of the clavicle) were performed on 1.5T or 3.0T MR imaging systems, using dedicated head and neck combined coils. Patients first underwent non‐contrast‐enhanced T1‐weighted image (T1WI) and T2‐weighted image (T2WI) scans in the axial, coronal, and the sagittal planes. Subsequently, the contrast agent gadolinium‐diethylenetriamine pentaacetic acid (Gd‐DTPA; 0.1 mmol/kg) was injected intravenously using an automatic high‐pressure injector and contrast‐enhanced T1‐weighted images were acquired. The scanning parameters for T1WI scans were fast spin‐echo (FSE), repetition time (TR) = 540 ms, and echo time (TE) = 11.8 ms. For the T2WI scan, the parameters were FSE, TR = 4000 ms, and TE = 99 ms. The section thickness was 5 mm and the intersection gap was 1 mm.

### 2.3 MRI assessment

All MR images were independently analyzed by two experienced radiologists. Disagreements were resolved by discussion and, if necessary, a third radiologist was consulted to help reach a consensus. As explained above, the 8th AJCC staging system differentiates between T2 and T3 stage NPC based on the presence or absence of invasion into the bony structures of the skull base or cervical vertebra and/or paranasal sinuses. In this study, however, instead of evaluating the invasion of the skull base as a whole, the radiologists assessed the invasion of each of the structures in the skull base. Table 1 lists the structures examined by the radiologists for signs of tumor invasion; the additional structures specifically examined in this study are in bold font. Invasion of the parapharyngeal space was defined as tumor extension beyond the pharyngobasilar fascia^15^ and invasion of the carotid sheath was defined as tumor extension into the post‐styloid space, as the carotid sheath region is medial to the styloid.^16^ Since invasion of other structures are obvious and easy to read on MRI images, they are not listed here.

**TABLE 1:** Structures examined for T staging in the AJCC 8th staging system and our staging system

Anatomic structures	Structures examined in the AJCC 8th version staging system	Structures examined in the proposed staging system^a^
**Soft tissue**	Tensor veli palatini muscle	Tensor veli palatini muscle
	Levator veli palatini muscle	Levator veli palatini muscle
	Longus capitis muscle	Longus capitis muscle
	Medial pterygoid muscle	Medial pterygoid muscle
	Lateral pterygoid muscle	Lateral pterygoid muscle
	Temporalis muscle	Temporalis muscle
	Parapharyngeal space	Parapharyngeal space
		**Left and right side for each soft tissue**
		**Pharyngeal recess**
		**Pterygopalatine fossa**
		**Carotid sheath**
		**Anterior segment of the styloid process**
		**Anterior intervertebral space**
		**Anterior lacerate fissure**
		**inferior orbital fissure**
**Skull‐base invasions**	Skull‐base invasions	**Base of sphenoid bone**
	Cervical vertebra	**Base of the pterygoid process**
		**Great wing of the sphenoid bone**
		**Clivus ossis occipitalis**
		**Petrous apex**
		**Base of the occipital bone**
		**Cervical vertebra**
**Paranasal sinuses**	Paranasal sinuses	Paranasal sinuses (sphenoid sinus ethmoid sinus, maxillary sinus, frontal sinus)

Abbreviation: AJCC, American Joint Committee on Cancer.

^a^ The additional structures examined for the proposed staging system are in bold font.

### 2.4 Treatment and follow‐up

All patients received intensity‐modulated radiation therapy (IMRT). Briefly, the prescribed dose to nasopharyngeal gross tumor volume (GTVnx), that is, the primary tumor seen on clinical examination and in radiographs, was 66‐72 Gy, and the dose to the metastatic lymph node area (GTVnd), that is, the clinically and/or radiologically observed enlarged lymph node area, was 64‐70 Gy. In addition to IMRT, patients (stage II‐IV) also received cisplatin‐based concomitant or induction chemotherapy. Details of IMRT planning and dose prescription have been described previously.^4^, ^17^, ^18^

Patients were followed up every 3 months in the first 2 years, and every 6 months thereafter, with a total follow‐up duration of 5 years. The endpoints were overall survival (OS), defined as the period from the date of initial diagnosis to the date of death due to any cause; local recurrence‐free survival (LRFS), calculated as the period from the date of initial diagnosis to the date of relapse; and progression‐free survival (PFS), defined as the period from the date of initial diagnosis to the date of relapse or death from any cause, whichever occurred first.

### 2.5 Statistical analyses

Survival analysis of T2 and T3 stage NPC patients was performed using the Kaplan‐Meier method and the log‐rank test was used to compare differences between groups. The recommended new T2 and T3 staging systems were developed in three steps. First, we performed network analysis to identify the factors that could be used for downstaging T3 stage NPC or upstaging T2 stage NPC. Network analysis is a method that characterizes the networked structures in terms of nodes and connects and visualizes the nodes with ties. Using OS as the outcome, we performed network analysis for the soft tissues (ie, AJCC T2 stage structures) and bony structures (ie, AJCC T3 stage structures). Second, we used Cox proportional hazards regression analysis to calculate the hazard ratios (HR) for death in the presence of tumor involvement of different soft‐tissue and bony structures, after adjusting for potential confounders (age, sex, HBV status, and pretreatment EBV load). We then ranked the HRs of each of the soft‐tissue and bony structures. Third, we selected two soft‐tissue structures with the shortest Euclidean distance from the death node in network analysis and with the largest HRs in Cox regression analysis for upstaging to T3. Similarly, we selected two bony structures with the shortest Euclidean distance from the survival node and the lowest HRs as the structures that could be used for downstaging to T2. We applied these criteria to reclassify T stage in patients in the training samples. Fourth, we compared the differences in the survival curves for OS, LRFS, and PFS between the reclassified T2 and T3 stage NPC patients using the log‐rank test. Finally, we validated the robustness of the modified T2 and T3 staging systems in the First People's Hospital of Foshan dataset. Statistical analysis was performed using R software (version 3.3.2; mice, rms, survival, Sizer, party libraries). All statistical tests were two‐sided and *P* < 0.05 was considered statistically significant.

## 3 RESULTS

### 3.1 Sociodemographic and clinical characteristics of participants

Table 2 shows the sociodemographic and clinical characteristics of the participants. In the training dataset (n = 792), 72.7% of the participants were male, 50.1% were ≥45 years old, 38.3% had stage III NPC, and 94.2% had WHO histological type 3. In the validation dataset (n = 455), 76.3% were male, 59.3% were ≥45 years old, and 38.0% had stage III NPC. Age, sex, T stage, and N stage were comparable between the training and validation datasets.

**TABLE 2:** Sociodemographic and clinical characteristics of the participants

Variables	Training sample (N = 792)	Validation sample (N = 433)	*P*‐value
Sex			0.212
Male	576 (72.7)	329 (76.0)	
Female	216 (27.3)	104 (24.0)	
Age			0.002
≥45 years old	397 (50.1)	257 (59.4)	
<45 years old	395 (49.9)	176 (40.6)	
Histologic type			<0.001
WHO type 1	5 (0.6)	—	
WHO type 2	41 (5.2)	—	
WHO type 3	746 (94.2)	433 (100)	
T classification			0.547
T1	204 (25.8)	121 (27.9)	
T2	97 (12.2)	60 (13.9)	
T3	296 (37.4)	146 (33.7)	
T4	195 (24.6)	106 (24.5)	
N classification			0.031
N0	182 (23.0)	75 (17.3)	
N1	438 (55.3)	241 (55.7)	
N2	113 (14.3)	84 (19.4)	
N3	59 (7.4)	33 (7.6)	
Stage			0.940
I	73 (9.2)	37 (8.5)	
II	175 (22.1）	100 (23.1)	
III	303 (38.3)	161 (37.2)	
IV	241 (20.4)	135 (31.2)	

Abbreviations: WHO, World Health Organization.

### 3.2 Invasion of skull structures and NPC prognosis

Figure S2 shows the results of network analysis for selecting potential up‐staging or downstaging factors. Invasion of the base of the sphenoid and the base of the pterygoid was very close to the survival node (Figure S2A), while the invasion of the bilateral parapharyngeal space or single/bilateral carotid sheath was close to the death node (Figure S2B). On multivariate Cox regression analysis, after adjusting for age, sex, HBV status, and pretreatment EBV load, AJCC T3 stage patients with the invasion of the base of the sphenoid (HR = 2.58, 95% confidence interval (CI) = 1.16‐5.77) or the base of the pterygoid (HR = 2.00, 95% CI = 0.84‐4.77) had significantly lower HRs than AJCC T2 stage patients with the invasion of bilateral parapharyngeal space (HR = 5.26, 95% CI = 2.02‐13.68) and single/bilateral carotid sheath (HR = 7.78, 95% CI = 3.06‐19.76; Table 3). Thus, it is reasonable to suggest that AJCC T3 stage NPC, with the invasion of the base of the sphenoid or base of the pterygoid, be reclassified as T2 stage, and that AJCC T2 stage NPC, with the invasion of bilateral parapharyngeal space or single/bilateral carotid sheath, be reclassified as T3 stage (Table 4).

**TABLE 3:** Associations between invasions of the cranial structures and NPC prognosis

Variables	Univariate Cox regression		Multivariate Cox regression		Multivariate Cox regression	
	HR (95% CI)^a^	*P*‐value	HR (95% CI)^b^	*P*‐value	HR (95% CI)^c^	*P*‐value
Structures for T2 stage						
Longus capitis muscle	3.24 (1.38,7.65)	0.007	3.44 (1.43,8.32)	0.006	1.41 (0.75,2.67)	0.285
Tensor veli palatini muscle	2.71 (1.17,6.29)	0.020	2.53 (1.08,5.92)	0.033	1.09 (0.60,1.97)	0.787
Medial pterygoid muscle	3.56 (0.94,13.42)	0.061	3.01 (0.77,12.81)	0.112	1.40 (0.41,4.78)	0.594
Single parapharyngeal space	2.36 (1.05,5.29)	0.038	2.28 (1.01,5.15)	0.048	1.10 (0.61,1.99)	0.743
Bilateral parapharyngeal space	4.79 (1.89,12.14)	0.001	5.26 (2.02,13.68)	0.001	1.61 (0.77,3.34)	0.204
Single/bilateral carotid sheath	5.91 (2.48,14.09)	0.000	7.78 (3.06,19.76)	0.000	3.23 (1.70,6.14)	0.000
Structures for T3 stage						
Base of sphenoid bone	2.71 (1.22,6.00)	0.014	2.58 (1.16,5.77)	0.021	1.50 (0.83,2.70)	0.174
Base of the pterygoid process	2.09 (0.89,4.92)	0.093	2.00 (0.84,4.77)	0.119	1.01 (0.54,1.89)	0.973
Petrous apex	3.24 (1.36,7.71)	0.008	3.58 (1.48,8.64)	0.005	1.71 (0.90,3.27)	0.103
Clivus ossis	3.27 (1.40,7.65)	0.006	3.35 (1.42,7.89)	0.006	1.55 (0.84,2.88)	0.163
Great wing of the sphenoid bone	5.40 (1.62,17.95)	0.006	6.67 (1.91,23.64)	0.003	2.38 (0.84,6.72)	0.102
Occipital and Cervical vertebra	4.30 (1.29,14.29)	0.017	7.93 (2.04,30.93)	0.003	1.92 (0.67,5.47)	0.222
Paranasal sinuses	6.75 (2.45,18.63)	0.000	8.39 (2.85,24.71)	0.000	2.99 (1.30,6.87)	0.010

No patient in the T2/T3 stage in our database had lateral pterygoid muscle invasion. Patients with T4 stage were excluded.

^a^ T1 stage was set as the reference group.

^b^ Adjusted for age, sex, HBV status (+/−), and pretreatment EBV level.

^c^ Adjusted for age, sex, HBV status (+/−), and pretreatment EBV level and N stage.

**TABLE 4:** Criteria for T stage classification by the 8th AJCC staging system and our staging system

T stage	AJCC 8th version staging system	Proposed new staging system
T2	Parapharyngeal extension	Single parapharyngeal extension
	Soft tissue involvement (medial pterygoid, lateral pterygoid, prevertebral muscles)	Soft tissue involvement (medial pterygoid, lateral pterygoid, prevertebral muscles)
		Invasion of the base of sphenoid bone or base of the pterygoid bone
T3	Bony structures (skull base, cervical vertebra) and/or paranasal sinuses	Bony structures (other skull base invasion, cervical vertebra) and/or paranasal sinuses; and/or bilateral parapharyngeal space; and/or single/bilateral carotid sheath area

### 3.3 Comparisons of survival between AJCC T2 and T3 stage NPC and the modified T2 and T3 stage NPC

**FIGURE 1:**
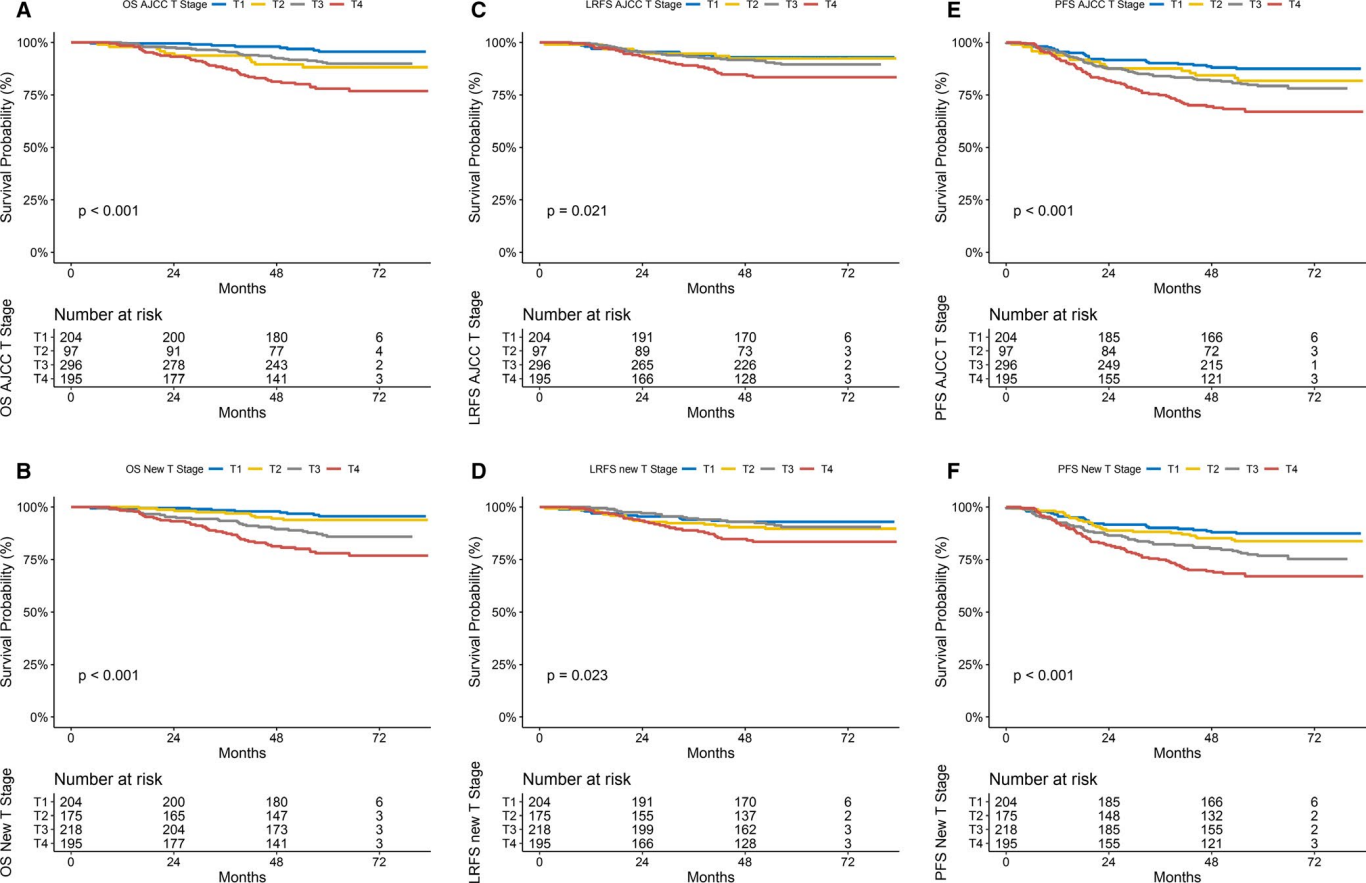
Survival curves of T1 to T4 stage NPC patients classified by the AJCC 8th version and our recommended new staging systems. The survival curves for OS of AJCC T2 and T3 stage NPC are close together (Figure 1A), whereas the survival curves for OS of reclassified T2 and T3 stage NPC are well separated from each other (Figure 1B). OS, overall survival; LRFS, local recurrence‐free survival; PFS, progression‐free survival

After applying our criteria, 100/296 (33.8%) AJCC T3 stage NPC cases were reclassified as T2 stage and 22/97 (22.7%) AJCC T2 stage cases were reclassified as T3 stage, which yielded a new sample containing 175 T2 stage and 218 T3 stage NPC cases. The 5‐year OS, LRFS and PFS were 88.2%, 92.4%, and 81.7% for the AJCC T2, and 89.9%, 89.5%, 79.3% for the AJCC T3 stage NPC, respectively, vs 93.9%, 89.7%, and 83.8% for the reclassified T2, and 85.9%, 76.8% for the reclassified T3 stage NPC, respectively. Figure 1 shows the survival curves (OS, LRFS, and PFS) of T1 to T4 stage NPC patients classified by the AJCC 8th version and by our modified staging system. The survival curves for OS of AJCC T2 and T3 stage NPC are close together (log‐rank test *P* = 0.531), whereas the survival curves for OS of reclassified T2 and T3 stage NPC are well separated from each other (log‐rank test *P* = 0.020). We found a similar trend in PFS analysis. However, the survival curves for LRFS were close together for AJCC T2 and T3 stage NPC and reclassified T2 and T3 stage NPC.

Figure S3 shows the N stage survival curves (OS, LRFS, and PFS) of T1 to T4 stage NPC patients classified by the AJCC 8th version.

### 3.4 Validation of our recommended new T2 and T3 staging systems

**FIGURE 2:**
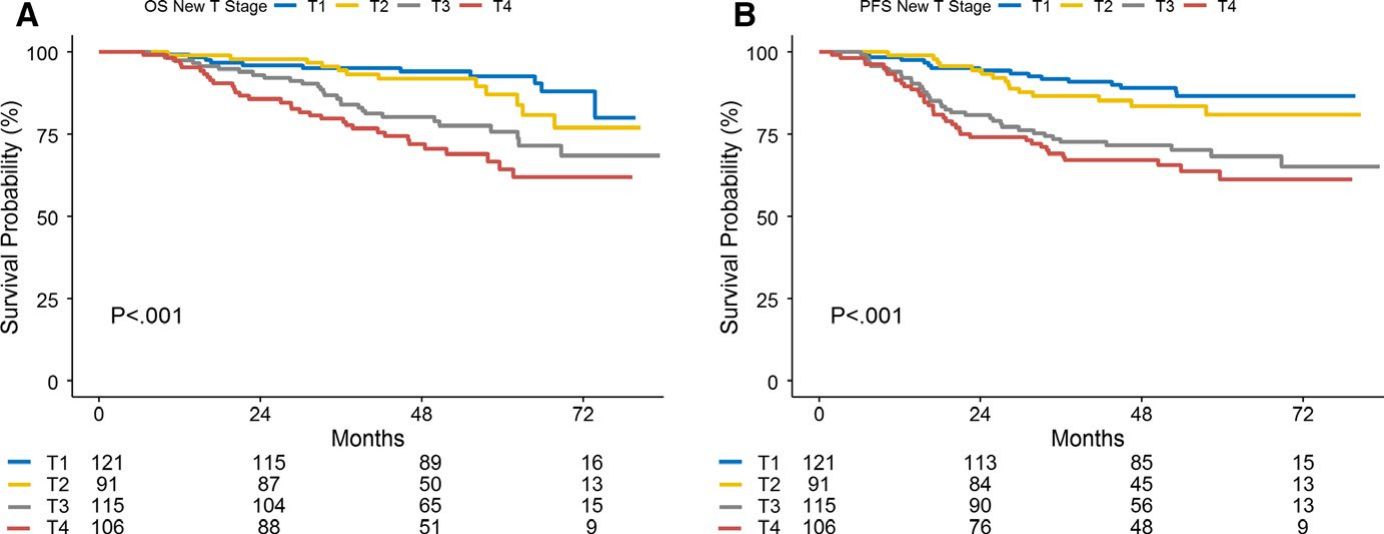
Survival curves of T2 and T3 stage NPC patients in the validation dataset classified by our recommended new staging system. The survival curves for OS and PFS were clearly separated by our new staging system. Abbreviations: OS, overall survival; PFS, progression‐free survival

Figure 2 shows the survival curves for OS and PFS of T2 and T3 stage NPC patients in the validation dataset classified by our recommended new staging system. As in the training dataset, the survival curves for OS and PFS were clearly separated by our recommended new staging system. The 5‐year OS and PFS were 87.2% and 75.7% for T2, and 75.7% and 68.1% for T3 stage NPC patients, respectively.

It should be mentioned that after reclassifying patients with the new criteria, the survival curves (especially for PFS) of T1 and T2 stages showed some overlap in the training dataset (*P* = .368).

## 4 DISCUSSION

Our study confirmed that the 8th AJCC staging system does not separate the survival curves of T2 and T3 stage NPC cases. However, when AJCC T3 stage patients with the invasion of the base of the sphenoid bone or base of the pterygoid bone were downstaged to T2, and AJCC T2 stage patients with the invasion of bilateral parapharyngeal space or single/bilateral carotid sheath were upstaged to T3, the survival curves of the recommended new T2 and T3 stages showed clear separation. The recommended new staging system was also validated on an external sample.

Consistent with our hypothesis, we found that the invasion of different bony skull‐base structures had a widely differing effect on the long‐term survival of NPC patients. The best prognosis was seen with the invasion of the base of the sphenoid bone and the base of the pterygoid bone. This may be because these two bony structures are relatively close to the nasopharynx and therefore more likely to be invaded in the early stages when the tumor is small and the prognosis better. Furthermore, these two bony structures are located within the pharyngobasilar fascia, which acts as a barrier to remote metastasis.^19^, ^20^ Once the tumor extends to the bony structures located outside the pharyngobasilar fascia, the risk of remote metastasis and death increases substantially. These findings are very similar to those of Chen et al,^9^ who observed that patients with the invasion of the base of the sphenoid or the base of the pterygoid had a better prognosis than those with the invasion of other bony structures, especially those with neural foramina. They suggested that instead of grouping all patients with skull base involvement together as one risk group, NPC patients should be classified into low‐risk and high‐risk groups according to the involvement of specific bony structures.

Consistent with previous reports,^15^, ^21^ we found that the invasion of the parapharyngeal space or single/bilateral carotid sheath was associated with poor prognosis. Parapharyngeal space involvement is known to be closely associated with distant metastasis,^16^, ^22^, ^23^ which would explain the poorer prognosis of these patients. An interesting finding was that PFS and OS were shorter with the invasion of bilateral parapharyngeal spaces than with the invasion of a single parapharyngeal space. This phenomenon indicated that the identification of bilateral parapharyngeal invasion would be useful for prognostication. We also found that patients with the invasion of single/bilateral carotid sheath (which is also called the post‐styloid parapharyngeal space) had poorer long‐term survival than those with the invasion of some of the bony structures. This is not surprising as carotid sheath invasion by the tumor is very likely to also involve the blood vessels within the carotid sheath,^8^ which would inevitably increase the risk of metastasis.

In this study, we attempt to create a recommended new staging system to separate T2 and T3 stage NPC cases. If this staging system is applied, about 30% of AJCC T2 or T3 stage NPC will be either upstaged or downstaged. The treatments for AJCC T2 and T3 stage NPCs differ, and this reclassification will help further optimize the treatment of NPC patients. Specifically, it will help avoid the overtreatment of some tumors that are misclassified as T3 stage NPC using the AJCC 8th edition. Moreover, it will also allow the timely intervention of other cases that are misclassified as T2 stage NPC.

The recommended new staging system described here has some limitations. First, it requires a thorough examination of MR images for evidence of invasion into each part, making the staging procedure more time‐consuming. Second, with this recommended new staging system, the survival curves for T1 and T2 stages show some overlap. More studies are needed to establish a system that can clearly separate the different T stages. Finally, it should be mentioned that our study sample was relatively small; therefore, studies on larger samples are needed to confirm our findings.

In summary, we propose a new T2 and T3 staging system for NPC based on the absolute effect sizes of invasion of each skull anatomical structure. The recommended new staging system clearly separates the survival curves of T2 and T3 stage NPC and could help improve the prognosis accuracy and treatment selection in NPC.

## CONFLICT OF INTEREST

There is no conflict of interest disclosed in this study.

## AUTHOR CONTRIBUTIONS

Conception and design: Chunyan Cui, Haojiang Li, Yue Yan, and Lizhi Liu. Financial support: Lizhi Liu. Provision of study materials or patients: Huali Ma, Jian Zhou, Annan Dong, Fei Xie, Shaobo Liang, Chuanbo Xie. Collection and assembly of data: Jian Zhou, Annan Dong, Fei Xie, Shaobo Liang, Li Li, Yue Yan. Data analysis and interpretation: Chunyan Cui, Haojiang Li, Huali Ma, Chuanbo Xie, Yue Yan, and Lizhi Liu. Manuscript writing: All authors. Final approval of manuscript: All authors.

## Supporting information

Fig S1

Fig S2

Fig S3

## Data Availability

The data analyzed during this study are available from the corresponding author on reasonable request.
