# Supplementary figures and images for: Staging of T2 and T3 nasopharyngeal carcinoma: Proposed modifications for improving the current AJCC staging system
Source: Cancer Med. 2020 Sep 1;9(20):7572–9. doi: 10.1002/cam4.3328 (PMC7571804; doi:10.1002/cam4.3328)

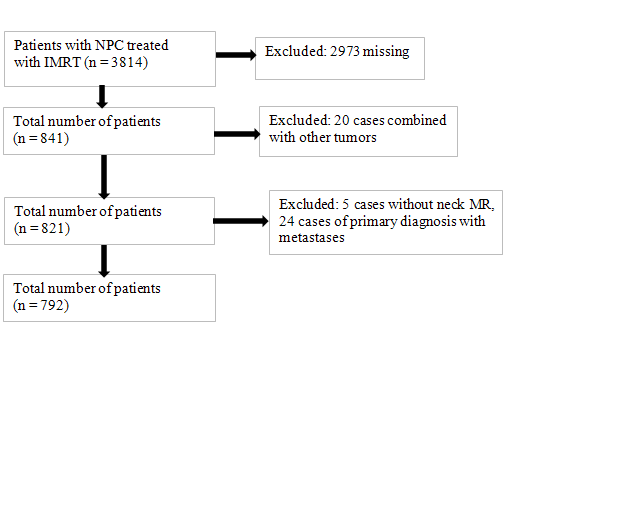

Supplement: Supplementary file 1 — Fig S1 [file CAM4-9-7572-s001.tiff]

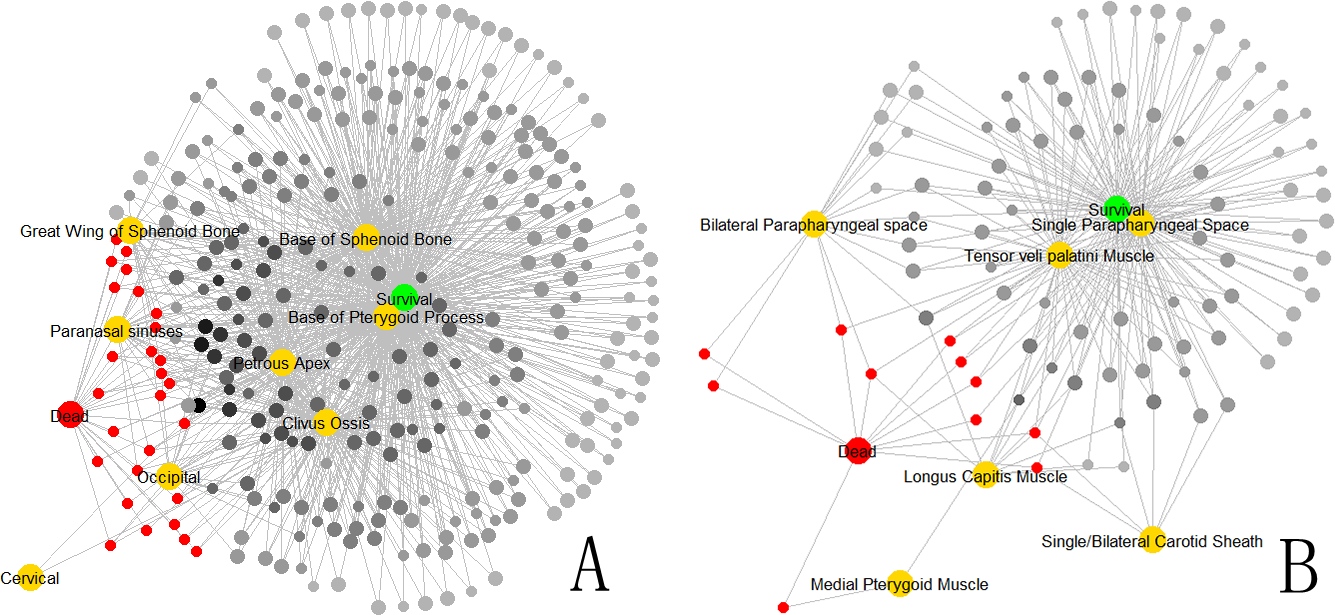

Supplement: Supplementary file 2 — Fig S2 [file CAM4-9-7572-s002.tiff]

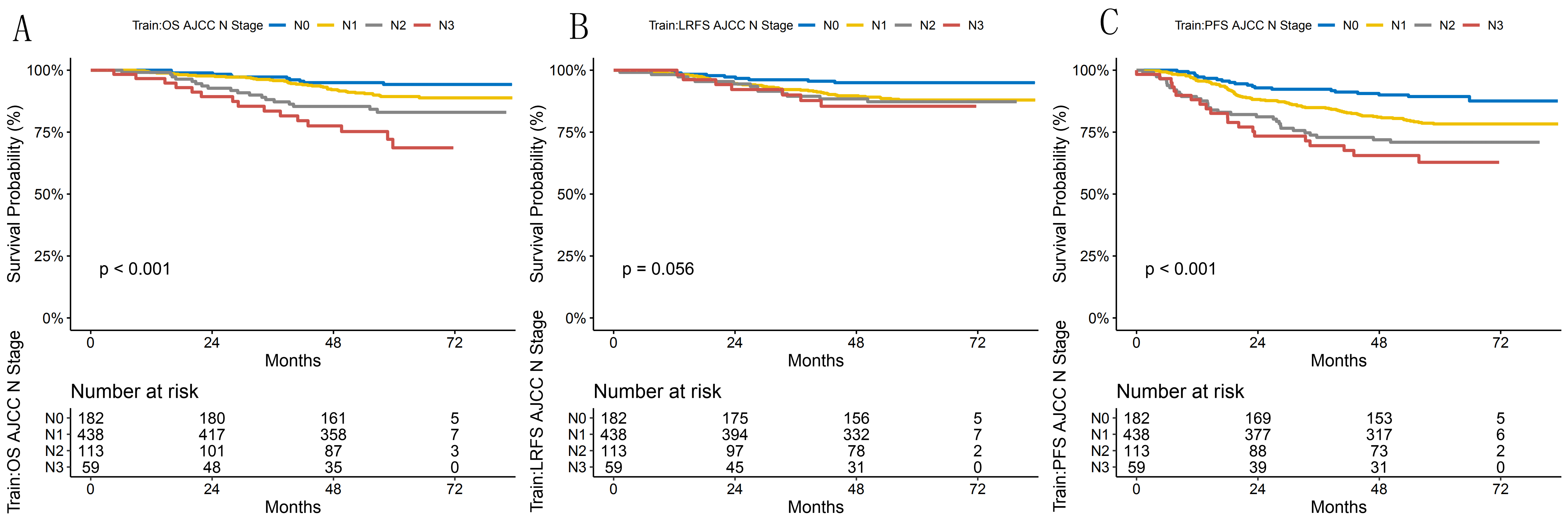

Supplement: Supplementary file 3 — Fig S3 [file CAM4-9-7572-s003.tiff]
